# Bioactive calcium phosphate–based glasses and ceramics and their biomedical applications: A review

**DOI:** 10.1177/2041731417719170

**Published:** 2017-07-21

**Authors:** Md Towhidul Islam, Reda M Felfel, Ensanya A Abou Neel, David M Grant, Ifty Ahmed, Kazi M Zakir Hossain

**Affiliations:** 1 Advanced Materials Research Group, Faculty of Engineering, University of Nottingham, Nottingham, UK; 2 Physics Department, Faculty of Science, Mansoura University, Mansoura, Egypt; 3 Division of Biomaterials, Operative Dentistry Department, Faculty of Dentistry, King Abdulaziz University, Jeddah, Saudi Arabia; 4 Biomaterials Department, Faculty of Dentistry, Tanta University, Tanta, Egypt; 5 Biomaterials and Tissue Engineering Division, Eastman Dental Institute, University College London, London, UK

**Keywords:** In vitro, bioactivity, glass, ceramic, calcium phosphate

## Abstract

An overview of the formation of calcium phosphate under in vitro environment on the surface of a range of bioactive materials (e.g. from silicate, borate, and phosphate glasses, glass-ceramics, bioceramics to metals) based on recent literature is presented in this review. The mechanism of bone-like calcium phosphate (i.e. hydroxyapatite) formation and the test protocols that are either already in use or currently being investigated for the evaluation of the bioactivity of biomaterials are discussed. This review also highlights the effect of chemical composition and surface charge of materials, types of medium (e.g. simulated body fluid, phosphate-buffered saline and cell culture medium) and test parameters on their bioactivity performance. Finally, a brief summary of the biomedical applications of these newly formed calcium phosphate (either in the form of amorphous or apatite) is presented.

## Introduction

There is a high need for smart off-the-shelf materials that are capable of confirming to the shape of tissues being replaced, modulating cellular function and promoting tissue regeneration. These materials could potentially provide morphological^1–3^ or biochemical cues^4,5^ that guide cellular interaction which is essential for tissue regeneration. Examples of these smart materials include bioactive glasses and ceramics that undergo specific surface reactions when incubated in simulated body fluid (SBF)^6^ or implanted in animal or human body^7^ leading to the formation of hydroxyapatite (HA) layer that forms a strong bond with the host tissues. They are osteoinductive and osteoconductive; therefore, they attracted much interest in bone tissue engineering.^8^ They can also be used for soft tissue regeneration^9^ and drug delivery applications.^10^

### Bioactive glasses

These glasses are amorphous and can be prepared either by melt–quench or sol–gel process.^11^ The sol–gel technology allows for incorporation of biomolecules, proteins or drugs that can be delivered in situ.^12,13^ Generally, these glasses can be tailored to release metallic ions that have antimicrobial^14,15^ or angiogenic potentials.^16,17^ They can also be prepared as solid or nano-/micro-/macro-porous scaffolds^18^ with highly ordered, controlled pore size and pores interconnectivity.^19^ The porous configuration is necessary for cell migration, angiogenesis and tissue infiltration; this further enhances the bond to the host tissues.^20^ Functionalisation of the scaffold with amino or carboxylic groups^21^ or loading anti-osteoporotic drugs (e.g. ipriflavone)^22^ could also be achieved to further improve bone regeneration. Microspheres can also be prepared^23^ using different techniques (flame spheroidisation,^24^ laser^25^ and thermally induced phase separation and/or oil-in water processing^26^) for various applications such as radiotherapy,^27^ drug delivery^22^ and tissue engineering.^26^

Under this category, silicate, phosphate and borate glasses will be discussed.

#### Silicate-based glasses

Silicate glasses are mainly based on SiO_2_ (the glass network former); other modifying oxides are also included as Na_2_O, CaO and P_2_O_5_ to adjust the properties of the produced glass. These oxides are usually included in specific molar ratios to produce a biologically active glass. To improve the bioactivity of these glasses, three important compositional features, including (1) SiO_2_ content <60 mol%, (2) high Na_2_O and CaO content and (3) high CaO/P_2_O_5_ ratio,^20^ must be fulfilled. During the glass preparation, a spontaneous crystallisation is undesirable as it reduces the rate of HA formation on its surface.^28^ The rate of HA layer formation is highly dependent on the degradation of glass. Accordingly, the presence of Na_2_O and other alkali or alkaline earth metals increases the rate of HA layer formation. The presence of high silica content or multivalent ions, for example, boron and aluminium, reduces the rate of glass breakdown and hence the apatite layer formation rate.^29^ Due to their texture (pore size/volume and high surface area), sol–gel-produced glasses showed higher bioactivity than the melt-quenched counterparts.^11^ Furthermore, using the sol–gel technique, the silica content can be increased from 60 mol% in melt-quenched to 85 mol% in sol–gel without reducing the bioactivity.^30^

A family of silicate-based glasses have been used for dental and orthopaedic applications^29^ since 1960s under the commercial name of 45S5 Bioglass™. This formulation has been used as a benchmark for measuring the properties of new silicate glass compositions. When doped with boron, 45S5 Bioglass showed angiogenic potential caused by their ionic dissolution products.^17,31^ Boron^32^ and silver^14^ also induced antibacterial action to bioactive silicate glasses.

Silicate-based glasses, however, require high melting temperature during their manufacture, and the addition of various metal oxides to reduce the melting temperature could adversely affect the glass bioactivity. The compositional range and form of this glass is also limited. The degradation of this glass often takes 1–2 years to totally degrade,^11^ and the long-term effect of silica is still questionable.^33^ Fabrication of fibrous scaffold from this glass is also difficult. The search for new bioactive materials that could overcome the limitations of silicate-based glasses has led to the emergence of phosphate and borate-based glasses as alternatives. These glasses can be easily formed without significant crystallisation during their preparation.

#### Phosphate-based glasses

This class of glasses is mainly based on P_2_O_5_ (the glass network former), Na_2_O and CaO. Other modifying oxides, for example, CuO,^15^ ZnO,^34^ Ag_2_O,^35^ Fe_2_O_3_,^36^ TiO_2_^37^ and SrO,^38^ can also be included to induce a specific property, function or different biological response.^39–41^ Unlike silicate glasses, the phosphate tetrahedral has one terminal oxygen; this reduces the network connectivity and hence the rigidity but increases the compositional range of the produced glasses.^42^ Unlike vitreous silica, P_2_O_5_ is chemically unstable; addition of metal oxides improves its stability.^43^ The degradation of these glasses varies from hours to years according to the composition and intended applications.

These glasses can be prepared in different forms including discs,^44–46^ microtubes,^47^ microspheres^24,48^ and fibres.^36,41,49^ Fibres can be used as cell transportation and expansion device,^48^ nerve conduit^50^ or as a scaffold for muscle regeneration.^9^ Fibres with antibacterial properties (e.g. copper-containing) can be produced as wound dressing meshes for the treatment of leg ulcers and severe burns.^15^ The phosphate glass fibres have an intriguing ability to form microtubes; therefore, they can be incorporated within various polymers to help in diffusion of nutrients and ingrowth of vascularisation when used as scaffolds for soft and hard tissue regeneration.^51^

Phosphate glass microspheres were also prepared^23^ for radiotherapy^27^ applications. The morphology of microspheres provides a stable surface for cells to attach and proliferate^24^ and prevent tissue damage and haemorrhage when used for radiotherapy.^27^ The spherical morphology would provide large interstitial spaces that can be consistent and quantifiable for cell growth and proliferation than randomly shaped particles when packed into perfusion bioreactors.^24^

#### Borate-based glasses

Using borate $\left({\mathrm{BO}}_{3}^{3-}\right)$ in the glass network provides faster degrading glasses with rapid and complete conversion into HA than silicate-based glasses.^8^ Controlling the boron content tailors the degradation rate of these glasses.^52^ Boron has also beneficial action on bone remodelling and repair.^53^ Furthermore, the presence of boron may reduce the possibility of bacterial infection through its antimicrobial action.^32^ An example of borate-based glasses is D-AlK-B (double alkali borate) glass, based on Na_2_O–K_2_O–MgO–CaO–SiO_2_–P_2_O_5_–B_2_O_3_ system.^54^

These glasses supported in vitro cell proliferation^55^ and in vivo tissue formation;^56^ they could also be used as drug delivery vehicles.^57^ However, the main concern with these glasses is their potential toxicity.^8^ The degradation products of certain concentration produced an inhibitory effect on the growth of goat bone marrow stromal cells. Adjusting the pH of the glass extract and reducing the concentration of boron to be less than 2.96 mM were observed to stimulate the cell proliferation. Adjusting the boron content to get a reasonable cellular response may jeopardise the bioactivity of these glasses.^54^ The toxicity could also be reduced by dynamic culture conditions.^58^

### Ceramics

Another class of bioactive materials include calcium phosphate (CaP)-based ceramics (i.e. crystalline materials), for example, HA, β-tricalcium phosphate (β-TCP) and biphasic CaP (a mixture of HA and β-TCP).

HA can be produced as solid or porous materials. The porous configuration with pores <10 µm in diameter is essential for circulation of body fluids and those >100 µm are essential for colonisation of target cells.^20^ HA is normally sintered above 1000°C in a granular or block form; after sintering, it cannot be reshaped (if they are present in block form) to fit the defect and it is non-degradable. β-TCP, however, is degradable. The degradation of biphasic CaP is highly dependent on the ratio of its components; the higher the β-TCP content, the faster the degradation. Generally, the degradation of CaP ceramics varies according to their type, porosity, surface area (granular vs blocks) and degree of crystallinity (high crystallinity means low degradation).^59^

Injectable CaP ceramics are also available. They can be easily delivered through a minimally invasive method into the defect as aqueous-based paste. They then set, fill the defect and support tissue regeneration over time.^60^ This allows for their use as drug delivery vehicle^61^ or treating a defect in challenging areas, for example, craniofacial complex^59^ or vertebroplasty.^62^ Examples of CaP ceramics that are commercially available include Norian^®^ (Synthes Craniomaxillofacial, USA), BoneSource^®^ (Stryker Leibinger, Germany) and Mimix^®^ (Walter Lorenz Surgical, USA).^59^

### Glass-ceramic materials

Glass-ceramics are partially crystallised glasses that are produced by heating the parent glass above its crystallisation temperature.^63^ Unlike spontaneous surface crystallisation, which is undesirable during glass manufacturing, the crystallisation process is controlled. As a result, the produced glass-ceramics contain one or more crystalline phases embedded in a residual glassy phase.^64^ The bioactivity of glass-ceramics is highly dependent on proportion and type of crystals formed during crystallisation process.^65^ Controlled crystallisation yields dense, strong materials with unusual combinations of properties when compared with their parent glasses.^66^ It is also possible to design glass-ceramics with nano- or micro-structure according to the end application.^64^

A common example of glass-ceramics is apatite/wollastonite (A-W) that has improved mechanical properties than their parent glass.^67^ Due to their micro-nanostructure and improved mechanical properties, these glass-ceramics could be promising matrices for bone regeneration,^68^ for example, intramedullary plug in total hip replacement.^69^ Surface functionalisation of glass-ceramics with lysine improved their cytocompatibility.^70^

Regardless of the most obvious advantages of these bioactive glasses and ceramics, their brittle nature remains a big challenge particularly with the production of porous scaffolds. The expected reduction in strength associated with the degradation of the scaffold is also another challenge that requires careful consideration during scaffolds’ designing.

## Mechanism of apatite formation

**Figure 1.**
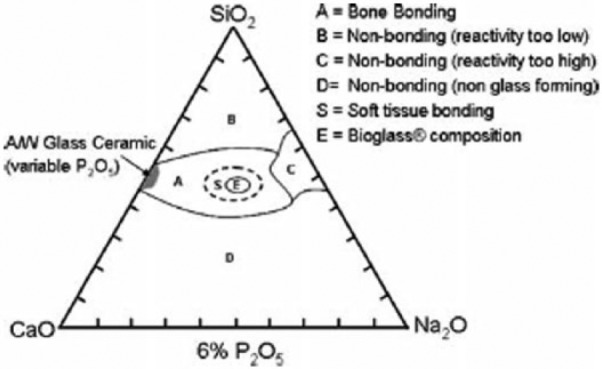
Compositional diagram representing the bone-bonding properties of bioactive glasses. Adapted with permission from Hench.^76^

Glass composition, surface charge, types of the medium (supersaturated solutions) and test conditions are the most influencing factors that affect the nucleation of apatite onto bioactive materials.^71^ The mechanisms of bioactivity for various bioactive materials (such as silicate, borate glasses and some metals) have been described in detail elsewhere.^52,71–75^ Among them, the mechanism of hydroxycarbonate apatite (HCA) layer formation on the surface of silicate-based glass (especially, 45S5 bioglass) implant has been most widely investigated. As the bioactivity of glasses mainly depends on the compositions of bioactive materials, a bone-bonding compositional diagram of silicate glass system (SiO_2_–CaO–Na_2_O–P_2_O_5_) has been proposed by Hench and colleagues^76,77^ (as presented in Figure 1). The diagram suggested that the glasses with composition fall within the region A are bioactive and hence can induce bonding with the bone, whereas compositions in region B are nearly bioinert. Compositions in region C are highly resorbable (10–30 days) and those fall within the region D do not form glass. Therefore, selection of proper compositions which in turn regulates the surface activity of the glass materials is important to understand the mechanism of apatite formation when tested in vitro to evaluate the in vivo bone-bonding capacity of bioactive materials. For example, a comparative study between in vivo bone ingrowth and in vitro apatite formation in SBF was investigated using Na_2_O–CaO–SiO_2_ glass system which reported that the induction period for apatite formation on the glass surface in SBF increased with increasing SiO_2_ content (from 50 to 70 mol%) which was well correlated with the results obtained from the in vivo bone ingrowth study.^78^

**Figure 2.**
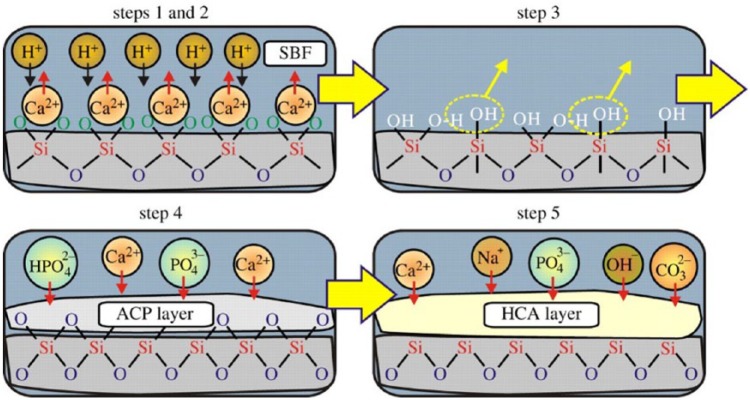
Schematic illustration of the reaction mechanism of HCA formation on the surface of silicate based bioglass according to Hench and colleagues.^79, 80^ Adapted with permission from Gunawidjaja et al.^81^

Apatite formation on the surface of bioactive glasses occurs through a sequence of chemical reactions when immersed in SBF. A schematic illustration of the reaction sequence leading to HCA formation according to Hench and colleagues^79,80^ has been described in Figure 2.^81^ First two stages involve the ion exchange reactions between the modifier ions of glass network (like Ca^2+^ and Na^+^) and H^+^ ions from the medium (SBF) which promote the hydrolysis of silica groups and followed by formation of silanol (Si–OH) groups on the glass surface. At stage 3, condensation and polymerisation of SiO_2_-rich layer take place on the surface, whereas stage 4 implies migration of Ca^2+^ and ${\mathrm{PO}}_{4}^{3-}$ ions from the glass network and also from SBF medium to the SiO_2_-rich layer leading to the formation of amorphous calcium phosphate (ACP) layer. At the final stage, uptake of additional ions from medium such as OH^−^, ${\mathrm{CO}}_{3}^{2-}$ and Na^+^ into the ACP layer promotes the conversion of ACP into HCA via crystallisation.

The release of calcium ions in combination with phosphorous ions was reported to help in deposition of apatite layer on the glass surface according to the mechanism mentioned above.^82,83^ For example, large amount of Ca^2+^ ion released from CaO–SiO_2_–TiO_2_ glass was found to form apatite layer on its surface within a day of immersion in SBF which was suggested to be due to increase of ionic activity during the apatite nucleation process.^84^ Several researchers also investigated the effect of Mg^2+^ ions on the bioactivity study and suggested that trace amounts of Mg^2+^ ions could enhance in vivo bone formation and adhesion of osteoblast cells on the glass surface.^85–91^ For example, MgO content up to ~17 wt% in MgO–3CaO·P_2_O_5_–SiO_2_ glass system was reported to promote CaP-rich layer formation and rapid mineralisation on the surface when immersed in SBF.^91^

**Figure 3.**
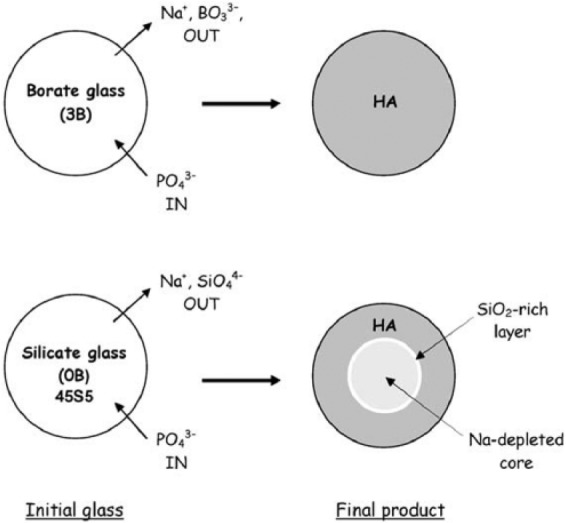
Schematic illustration of the mechanisms of conversion of borate (3B: B_2_O_3_-46.1, CaO-26.9, Na_2_O-24.4, P_2_O_5_-2.6 in mol%) glass and 45S5 (0B: SiO_2_-46.1, CaO-26.9, Na_2_O-24.4, P_2_O_5_-2.6 in mol%) glass to HA in a dilute phosphate solution. Adapted with permission from Huang et al.^74^

Borate-based bioactive glasses (e.g. 46.1B_2_O_3_–24.4Na_2_O–26.9CaO in mol%) follow the same mechanism of HA layer formation as described for silicate-based glasses except for the formation of SiO_2_-rich layer.^73,74^ The faster dissolution rate of the borate glass when compared to the silicate glass (due to their lower chemical durability) is considered as the main reason behind the fast deposition rate of HA-like material on surfaces of borate glasses.^52,74^ The conversion mechanisms of borate glass into HA in phosphate solution are illustrated in Figure 3.^74^ When borate glasses are immersed in a dilute phosphate solution, dissolution of Na^+^ and ${\mathrm{BO}}_{3}^{3-}$ ions from the glass structure into the solution occurs first. Then, ${\mathrm{PO}}_{4}^{3-}$ ions from the medium are assumed to react with Ca^2+^ ions leading to nucleation and growth of HA. The process is supposed to be continued until the glass is completely converted to HA.

**Figure 4.**
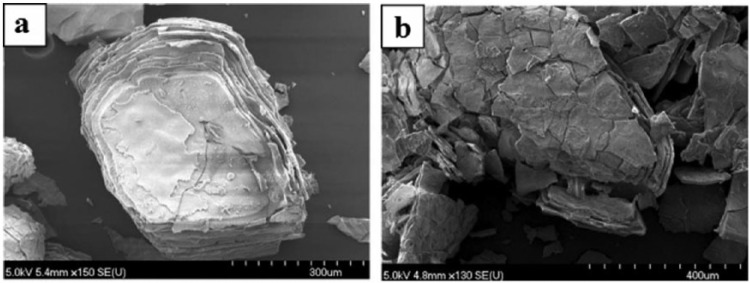
SEM images showing the reaction products for (a) silicate and (b) borate glasses after immersion in dilute K_2_HPO_4_ solution (20 mM). Adapted with permission from Huang et al.^74^

A comparative study on bioactivity of a borate (45B5) and silicate (45S5) glasses was carried out by Liang et al.,^92^ where borate glass was found to react faster (more than five times) than silicate glasses in a solution of 0.25 M K_2_HPO_4_ (pH = 9). HA layer was seen to form on borate glass within 24 h, whereas HA layer was not visible on silicate glass even after 7 days of immersion in the same medium. In addition, the conversion of borate glass into HA layer was reported to be completed within 4 days (in 20 mM K_2_HPO_4_ solution), while silicate glass (45S5) was seen to convert to HA partially (~50%) after 70 days of test period.^74^ For example, HA reaction product formed on the surface of silicate (SiO_2_-46.1, CaO-26.9, Na_2_O-24.4, P_2_O_5_-2.6 in mol%) and borate (B_2_O_3_-46.1, CaO-26.9, Na_2_O-24.4, P_2_O_5_-2.6 in mol%) glasses after immersion in dilute K_2_HPO_4_ solution (20 mM) had a layered structure (as presented in Figure 4(a) and (b)).

Bioceramics such as wollastonite and pseudowollastonite have been revealed faster apatite formation in SBF compared to other bioglasses and glass-ceramics.^93–96^ The mechanism of apatite formation on wollastonite in SBF was suggested to be due to the negative surface charge of the functional group (=Si–O–) on the ceramic surface rather than the dissolution of calcium ions into SBF.^97^ Various types of sintered bioceramics such as HA and α-tricalcium phosphate (α-TCP) were seen to form apatite layer on their surface after 24 h when immersed in SBF.^98^ The effect of pH (6.5 and 7.4) and the concentration of ${\mathrm{HCO}}_{3}^{-}$ ions (4.2 and 27 mM) in modified SBF (m-SBF) were also reported to have influence on the bioactivity response of bioceramics (e.g. commercial HA Captal^®^).^99^ No mineralisation was detected on the HA surface when tested in m-SBF at the pH of 6.5. However, HCA layer was seen to form on the HA surface in m-SBF at pH 7.4 and a thicker HCA layer was observed on the HA substrate that immersed in m-SBF containing higher ${\mathrm{HCO}}_{3}^{-}$ ions.

**Figure 5.**
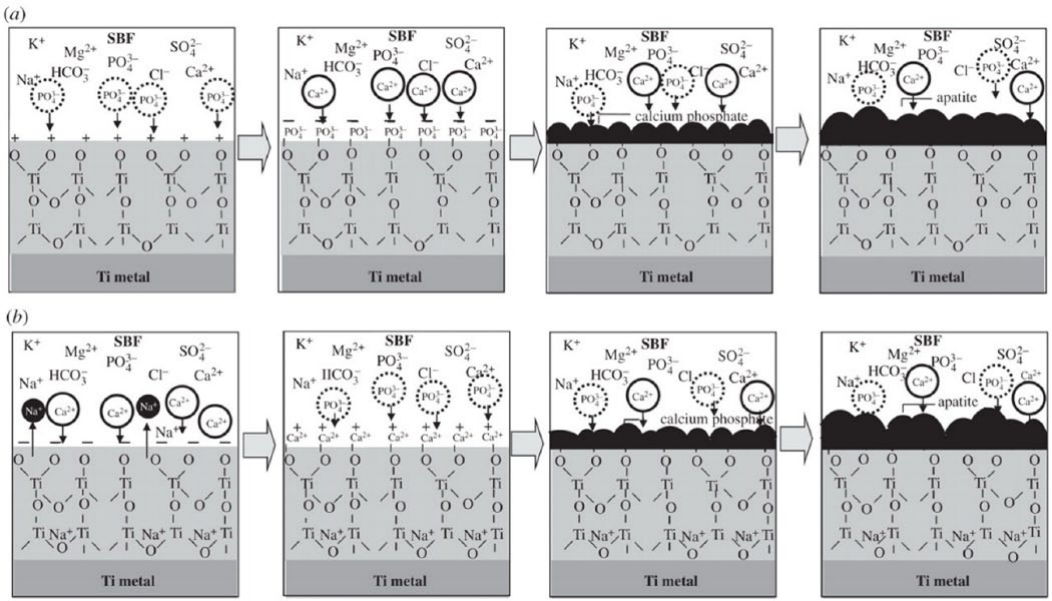
Schematic of ion adsorption on (a) positively charged and (b) negatively charged Ti metal in SBF medium. Adapted with permission from Pattanayak et al.^100^

In case of metal substrate (e.g. bioinert titanium, Ti), the surface charge and the pH of the medium play the vital role in the apatite formation.^72,100,101^ It has been previously reported that Ti and its alloys were found to form apatite when treated with basic (NaOH)^101,102^ or acidic (H_2_SO_4_/HCl) solution.^72^ Ti and its alloys display a certain level of positive and negative zeta potential when exposed to a strong acidic or basic solution, followed by a subsequent heat treatment. A schematic illustration of apatite formation mechanism on the positively and negatively charged Ti metal is described in Figure 5.^100^ When the positively charged Ti substrate is immersed into SBF, the negatively charged phosphate ions from the medium are assumed to first accumulate on its surface leading to creation of negatively charged surface. As a result, positively charged calcium ions (from medium) are migrated to the negatively charged surface in order to form a CaP layer prior to crystallisation into apatite layer, as shown in Figure 5(a). On the other hand, the Ti surface is expected to form a sodium titanate layer after alkali (in NaOH) and heat treatments. Afterwards, the sodium titanate exchanges Na^+^ ions with the H_3_O^+^ ions (from SBF) to form Ti–OH on the surface which leads to an increase in pH due to the consumption of H_3_O^+^ ions from medium and releasing Na^+^ ions to the medium. Consequently, Ti metal that carries a negative charge would initially attract calcium cations followed by phosphate anions to form CaP layer, as shown in Figure 5(b). Therefore, Ti–OH on the surface seems to induce apatite nucleation.^101,103^ For example, the bone-like apatite layer was reported to form on the NaOH (5M) and heat (600°C for 1 h)-treated Ti metal after immersion in SBF for 10 days. Likewise, alkali-treated titanium-based alloys such as Ti-6Al-4V, Ti-6Al-2Nb-Ta and Ti-15Mo-SZr-3Al were also reported to promote bone-like apatite deposition on their surfaces in SBF following the same mechanism (see Figure 5).^102,104^ Apatite formation on the surface of titanium has also been enhanced after acid treatment^105,106^ as well as by producing a negative surface charge via the light illumination of SBF with mercury lamp.^8^ Ti substrates without any treatment possess no surface charge; therefore, when exposed to SBF, they can only form a CaP layer on their surface which does not bond to the bone.^107^

Phosphate-based glasses (PBGs) have attracted huge interest in the field of biomaterials and tissue engineering due to their chemical similarity with the inorganic component of the natural bone and controllable degradation profile.^108^ However, very few literatures have examined the bioactivity of PBGs.^109–112^ The presence of TiO_2_ in phosphate glasses is reported to induce CaP nucleation and improve their bioactivity.^113^ Ti-doped PBGs were investigated for bioactivity and an intermediate hydrated titania layer (0.5–2 µm Ti–OH layer/gel) was observed to form in SBF which played an important role in the formation of apatite.^111^ Another bioactivity study on xCaO–(90-x)P_2_O_5_–10TiO_2_ glasses was suggested that the formulations containing 35 and 40 mol% of P_2_O_5_ did not support any apatite layer deposition even after 30 days of immersion in SBF. On the other hand, apatite layer was found to form on the phosphate invert glasses, containing 30 mol% of P_2_O_5_, after 20 days of immersion in SBF.^114^ This was suggested to be due to the release of relatively small amount of phosphate ions from lower P_2_O_5_ containing glasses which promoted the apatite nucleation.^109^

Apatite formation also depends on the basicity of the gel layer formed on the glasses and the amount of the functional groups present for HA nucleation in the layer.^114^ The basicity of the gel layer can be enhanced via the addition of Na_2_O.^109^ The high amount of Na_2_O and CaO as well as the relatively higher ratio of CaO/P_2_O_5_ would provide highly reactive surface of bioactive glasses in physiological environment which would eventually facilitate apatite formation.^115^ Hydrated gel layers such as Si–OH, Ti–OH, Ta–OH, Zr–OH, Nb–OH, –COOH and PO_4_H_2_ groups are proved to provide nucleation sites for HA in SBF.^116^

Apart from apatite formation, several other possible phases of calcium orthophosphates such as ACP, brushite CaHPO_4_·2H_2_O (DCPD), monetite CaHPO_4_(DCPA) or octacalcium phosphate (OCP) can be formed in SBF depending on the experimental conditions of formation and state of ageing.^117^ These calcium orthophosphates except ACP are more stable than HA in acidic conditions^118^ and at the later stages, all of the phases would be converted into HA in various pathways.^119^

## Test protocols for in vitro bioactivity experiment

In vivo bioactivity of a material can be predicted from its ability to form apatite in SBF and/or other similar types of supersaturated medium.^6^ A vast number of work have been focused so far on the effect of composition and morphology of glass and ceramic materials on their in vitro dissolution rate and apatite-forming ability. Various protocols with respect to the types of medium (e.g. SBF, phosphate-buffered saline (PBS), TRIS, K_2_HPO_4_, Dulbecco’s Modified Eagle’s Medium (DMEM)), morphology of materials (powder, pellets, discs), surface area to volume ratio and the test condition (static, dynamic) have been considered for the in vitro bioactivity experiment (summarised in Table 1).

**Table 1.** Parameters and test protocols that have been used to investigate the bioactivity of various biomaterials.

Materials	Medium	Geometry	Surface area or mass/vol	Condition	Length of study	Comments	Ref
Silicate glass (45S5)	SBF	Block (1 × 1.5 × 0.2 cm^3^)	S/V = 0.05 cm^−1^	Static	14 days	HA formed (2 days)	Helebrant et al.^120^
Glass-ceramics (A-W)	SBF	Block (22 × 40 × 2 mm^3^)	200 mL	–	30 days	HA formed (7 days)	Kokubo et al.^121^
Silicate glass (45S5/S53P4/S68)	SBF/TRIS/Na-PBS	Block (20 × 15 × 1.5 mm^3^)	20 mL S/V = 0.4 cm^−1^	Solution was replenished after 7 days	14 days	CaP layer formed on 45S5/S53P4 (24 h) and S68 (7 days)	Varila et al.^122^
Borate glass 15Na_2_O–15CaO–xB_2_O_3_–(70-x)P_2_O_5_	SBF	Particles (106–180 µm)	500 mg in 50 mL	–	30 days	HA formation increased with increasing B_2_O_3_ content	Abo-Naf et al.^123^
Silicate glass (37CaO–58SiO_2_–5P_2_O_5_)	SBF	Powder (<20 mm particles)	600 mg in 1 L	Stirring (100 r/min)	30 days	HCA formed (24 h)	Turdean-Ionescu et al.^124^
Silicate glass (46S6)	SBF	Disc (13 mm diameter × 5 mm thick)	30 mL	Agitation	30 days	HA formed (1 day)	Bui et al.^125^
Bioceramics (A-W; BG; HA; HA/TCP; α-TCP; β-TCP)	SBF	Cube (5 × 5 × 5 mm^3^)	200 mL	–	–	OCP formed (1 day) on all bioceramics except on β -TCP	Xin et al.^98^
Calcium aluminate (CA), glass ionomer cement (GIC), CA/GIC hybrid	PBS	Block (22 × 15 × 4 mm^3^)	50 mL	PBS was changed once a week	28 days	HA formed on CA (24 h) and CA/GIC hybrid (7 days) GIC did not show any bioactivity in PBS	Lööf et al.^126^
Silicate glass 41.7SiO_2_-(44.14-X) CaO-XMgO-3.13ZnO-5.2Na_2_O-K_2_O-4.7P_2_O_5_	SBF and Tris-buffer	Powder (<45 μm)	75 mg in 50 mL	Agitated using mechanical shaker	30 days	HA formed on non-magnesium containing glasses by 7 days in both SBF and Tris-buffer whereas HA formed on Mg containing glasses after 1 month in SBF but not in Tris-buffer	Al-Noaman et al.^127^
Bioceramics (HA)	SBF	Particles (<5 mm)	50 mg in 120 mL	–	120 h	HA formed (12 h)	Kim et al.^128^
Glass-ceramics (A-W)	SBF	Block (22 × 40 × 2 mm^3^)	200 mL	–	60 days	HA formed (7 days)	Kokubo et al.^129^
Glass-ceramic (Ceravital)	SBF	Block (15 × 10 × 1 mm^3^)	35 mL	–	20 days	HCA formed (1 day)	Ohtsuki et al.^130^
Borate glass (45B5)	K_2_HPO_4_ (0.25 M)	Disc (15-mm diameter and 3-mm thick)	–	–	14 days	HA formed (1 day)	Liang et al.^93^
Borate glass (36–61 mol% B_2_O_3_)	SBF/K_2_HPO_4_ (0.25 M)	Powder (25−75 μm)	1.5 mg/mL ratio	Gentle agitation	7 days	HCA formed (6 h)	Lepry and Nazhat^131^
Borate glass	K_2_HPO_4_ (0.2 M)	Disc (5-mm diameter × 5-mm thick)	100 mL	Static	7 days	HA formed (6 days)	Liang et al.^132^
Silicate glass (58S)	DMEM	Particles (20–40 µm)	75 mg in 50 mL	Solution was changed at 6 h, 24 h, and 2 days	3 days	HCA formed (3 days)	Theodorou et al.^133^
Titanium alloy (Ti6Al4V)	DMEM	Block (10 × 10 × 1 mm^3^)	40 mL	–	360 h	HA formed (360 h)	Faure et al.^134^
ISO/23317:2014(E)	SBF	Disc (10-mm diameter × 2-mm thick). Block (10 × 10 × 2 mm^3^)	Vs = Sa/10 mL ratio	Static	30 days	Apatite formation	ISO 23317:2014^135^
Unified method (TC04)	SBF	Particles (45–90 µm)	75 mg in 50 mL	Agitation (120 r/min)	28 days	Apatite formation	Maçon et al.^136^

SBF: simulated body fluid; HA: hydroxyapatite; PBS: phosphate-buffered saline; CaP: calcium phosphate; HCA: hydroxycarbonate apatite; DMEM: Dulbecco’s Modified Eagle’s Medium; OCP: octacalcium phosphate; TCP: tricalcium phosphate.

The effect of different solutions on dissolution of bioactive materials has been studied.^137–141^ In the early 1980s, TRIS buffer was used to evaluate the apatite-forming ability of glass and glass-ceramic materials.^142–144^ Later, in 1990, Kokubo et al.^121^ developed the simulated solution which reproduced in vivo surface structure changes of glass-ceramics A-W more precisely than TRIS. The pH of simulated solutions is maintained using TRIS of HEPES buffers. However, these buffers were seen unable to maintain the neutral pH of SBF during in vitro test.^140^ SBF is a supersaturated solution containing similar ionic concentrations of inorganic parts of human blood plasma (presented in Table 2).^145^ However, it has higher Cl^−^ ions and lower ${\mathrm{HCO}}_{3}^{-}$ ion concentration than those of the blood plasma. In 2001, Helebrant et al.^120^ investigated the apatite formation on 45S5 bioglass using a series of SBF solutions with increasing ${\mathrm{HCO}}_{3}^{-}$ ion concentration up to the value close to blood plasma and suggested that the SBF with increased amount of ${\mathrm{HCO}}_{3}^{-}$ ions is more appropriate for in vitro bioactivity testing of biomaterials. Oyane et al.^146^ also prepared the revised SBF (r-SBF) and modified SBF (m-SBF) which contained the ion concentrations equal or close to those in blood plasma (except for ${\mathrm{HCO}}_{3}^{-}$ ion concentrations in m-SBF).^147^ In terms of stability, r-SBF and m-SBF were seen to remain stable, no change in ion concentrations and pH value, up to 2 and 8 weeks, respectively, when stored in sealed containers at 36.5°C.

**Table 2.** Ionic concentration in human blood plasma in comparison with various developed SBF medium.^145^

Ion	Human blood plasma (pH 7.2–7.4)	Ion concentration (10^−3^ mol) in		
		SBF (pH 7.4)	Revised-SBF (r-SBF)	Modified-SBF (m-SBF)
Na^+^	142.0	142.0	142.0	142.0
K^+^	5.0	5.0	5.0	5.0
Mg^2+^	1.5	1.5	1.5	1.5
Ca^2+^	2.5	2.5	2.5	2.5
Cl^−^	103.0	147.8	103.0	103.0
${\mathrm{HCO}}_{3}^{-}$	27.0	4.2	27.0	10
${\mathrm{HPO}}_{4}^{2-}$	1.0	1.0	1.0	1.0
${\mathrm{SO}}_{4}^{2-}$	0.5	0.5	0.5	0.5

SBF: simulated body fluid.

In addition to SBF, cell culture medium (DMEM) were also used for bioactivity testing, and it has been found that the non-buffered DMEM solution containing an organic phase was not suitable for bioactivity test.^148^ DMEM contains lower concentration of Ca^2+^ ions but higher concentration of ${\mathrm{HCO}}_{3}^{-}$ ions compared to blood plasma which leads to formation of CaCO_3_ instead of apatite.^148^ However, recently Popa et al.^149^ studied the in vitro bioactivity of BG films (SiO_2_ 38.5, CaO 36.1, P_2_O_5_ 5.6, MgO 15.2, ZnO 4, and CaF_2_ 0.6 in mol%) in different medium (namely SBF, DMEM, DMEM supplemented with 10% foetal bovine serum) and found that bioactivity test in DMEM supplemented with proteins under homeostatic conditions was more appropriate than that in SBF. They also suggested an unique bioactivity testing protocol utilising specific surface area to medium volume ratio (Sa/V = 0.5 cm^2^/mL) for the materials with different shapes and dimensions including bulk objects, thin films, powder and scaffolds.^149^

PBS medium was also used in in vitro studies for bioactive glass (45S5) containing polymer (poly-l-lactic acid, PLLA and polylactic-*co*-glycolic acid, PLGA) composites.^150,151^ It was found that the formation of apatite on the glass surface was faster in PBS than SBF or TRIS.^139^ Fagerlund et al.^139^ investigated the dissolution of bioactive glasses (45S5, S53P4, 13-93) in PBS and reported that pH of the solution increased when alkaline and alkaline earth ions dissipated from the glasses. The release of silica ions and CaP precipitation also increased at higher pH. They also found that the CaP layer formed quickly due to the higher concentration of phosphorus ions in PBS.^139^ In vitro bioactivity of glasses especially for borate glasses has been evaluated in aqueous K_2_HPO_4_ medium.^57,92,131,152^ This medium was used to save the experimental time through the reaction of available ${\mathrm{HPO}}_{4}^{2-}$ and OH^−^ ions in K_2_HPO_4_ solution with glasses.^92^

Apart from the use of various media, other factors such as geometry of the test specimen, surface area to volume ratio and test conditions are also key to justify the test protocol. Therefore, an ISO standard (ISO/23317:2014(E): Implants for surgery – In vitro evaluation for apatite-forming ability of implant materials) has been proposed to conduct the in vitro bioactivity test.^135^ The ISO standard suggested to use acellular and protein-free SBF solution buffered with TRIS. The standard also suggested the dimension and shape of test specimen only for bulk compact inorganic materials (solid disc and rectangular block) with a defined sample surface area to SBF volume ratio (V_SBF_ = 100 × S_a_; where V_SBF_ is the volume of SBF and S_a_ is the surface area of glass). However, this ratio is not defined for the materials in other forms of biomaterials such as powder and porous scaffolds. Moreover, the ISO standard stated the static testing condition, whereas some literatures conducted dynamic condition to mimic the in vivo environment.^153–156^

Recently, the members of the Technical Committee 4 (TC04) of the International Commission on Glass (ICG) have been proposed unified method (modified version of ISO standard) for testing the bioactivity of glass particles (45–90 μm), particularly those of high surface area.^136^ The modified method suggested use of fixed mass per solution volume ratio (75 mg in 50 mL) with agitation (120 r/min).

## Biomedical applications of CaP

Earlier in section ‘Mechanism of apatite formation’, it has been mentioned that during the initial stage of the in vitro bioactivity study, the ACP layer formed onto the surface of bioactive materials followed by crystallisation into apatite (HA), which is nucleated by the interaction of ions and pH of the medium. However, some biomaterials can be limited to release of the desirable ions (due to their compositions) and also may be unable to produce favourable pH environment for ACP layer to be crystallised into apatite at the late stages of the in vitro study. Therefore, the biomedical applications of both ACP and apatite will be discussed in this section.

**Figure 6.**
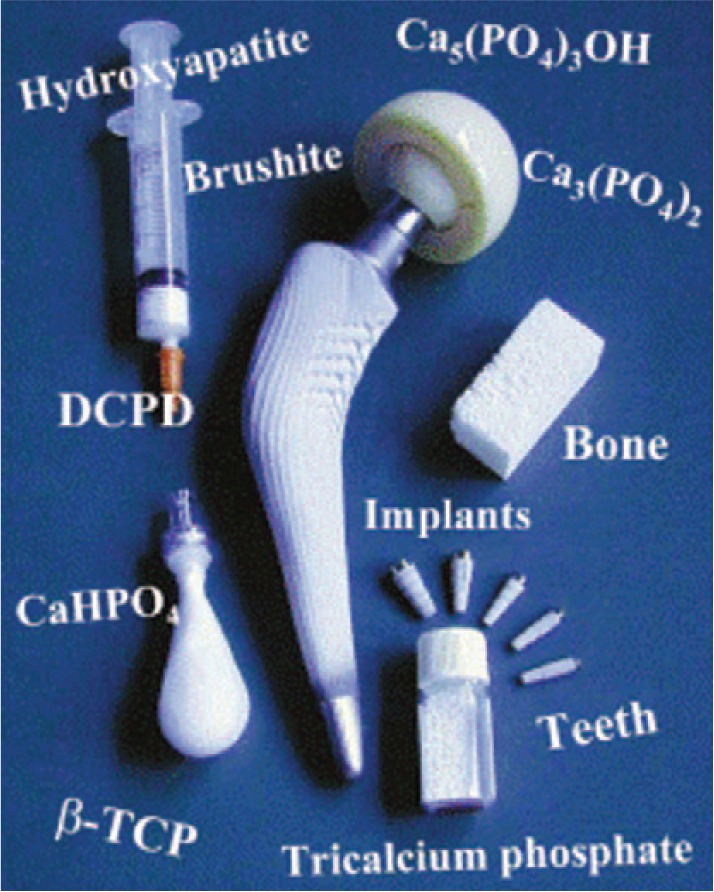
Examples of biomedical applications of CaP based materials (e.g. β-tricalcium phosphate, dicalcium phosphate, dicalcium phosphate dehydrate, tricalcium phosphate and calcium apatite) used in form of coating for hip prostheses and dental screws, porous bone graft, bone cements and pastes. Adapted with permission from Dorozhkin et al.^159^

Similar to apatite (i.e. HA), ACPs have excellent biological, osteoconductivity and no cytotoxicity responses; therefore, they have been introduced to orthopaedics and dentistry.^157^ ACPs have been investigated for a range of biomedical applications in different forms: powders, granules, composites, self-setting cements or coatings.^158^ Examples of biomedical applications of CaP-based materials can be seen in Figure 6.

### Dental applications

Due to good osteoconductivity and tuneable degradation rate of ACPs, they have been added to mouthwashes, chewing gums, toothpastes and also to ionomer cements as a filler for carious lesions. Complexes of casein phosphopeptides (CPP) and ACP have been used as abrasive pastes for treatment of tooth sensitivity after root canal repair, scaling or bleaching procedures. Clinical trial revealed an increase in the content of inorganic phosphate and calcium in supragingival plaque after 3 days’ use of mouthwash containing CPP-ACP complexes. This product is commercially known as GC Tooth Mousse. CPP-ACP complexes can be also incorporated into food, drinks and confectionary for potential prevention of the dental caries due to their natural origin (milk derivative). ACP has also been explored as a filler for bioactive polymer composites to be used for tooth repair. Tooth repair can be stimulated through the released ions (calcium and phosphate) and form the breakdown of ACP particles. Effect of ACP addition to orthodontic adhesives has also been evaluated and demonstrated satisfactory bracket bonding strength in clinical trials.^157^ Toothpastes containing CaP can be used to reduce tooth sensitivity, to promote remineralisation of the demineralised enamel and for whitening and polishing purposes. Toothpastes containing HA have shown a significant positive effect on sensitivity and whitening of tooth, and it was found that the whitening effect increased as the amount of HA within the toothpaste increased. Both ACP and HA have been added to toothpaste and are available commercially. Examples of HA-containing toothpastes are Sensitive Reminx (Pharma Jenistec Co., Ltd, Korea), Triple Denta (TripleLife Co., Ltd, Korea), Kalident – calcium hydroxyapatite (Kalichem Italia S.r.l.), DIO (DIO Co., Ltd, Korea), Coolin Bubble (Canavena Co., Ltd, Korea) and YP Dental (You Co., Ltd, Japan). There are also toothpastes containing ACP such as Enamelon, Arm and Hammer’s Enamel Care and Premier Dental’s Enamel Pro.^160^

### Bone repair

**Figure 7.**
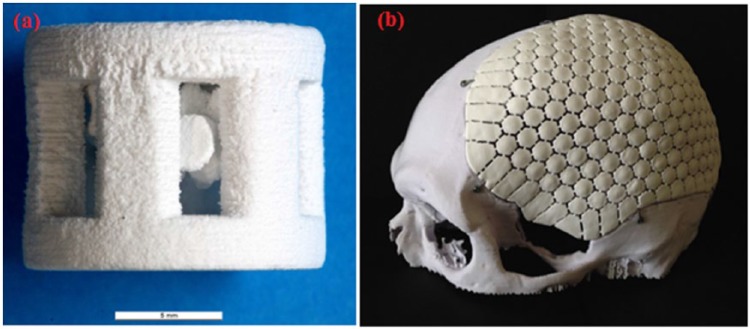
Examples of additive manufactured implants based on CaP; (a) 3D scaffolds of DCPA/monetite (scale bar: 5 mm), Adapted with permission from Butscher et al.^162^ (b) implant made of DCPA for treatment of cranial bone defects (Craniomosaic). DCPA is dicalcium phosphate. Adapted with permission from Habraken et al.^161^

Human bone contains 70% of CaP minerals; hence, CaP-based materials have been considered as the best choice for repairing the damaged bone post trauma.^161^ CaP has been thoroughly studied in various forms for repairing hard tissue because of their excellent biocompatibility, composition similarity with the bone mineral, inexpensiveness and easy to produce.^161^ It has been reported that the rate of new bone formation was well correlated with the rate of ACP resorption. Moreover, ACP revealed significantly better osteoconductivity response compared to TCP in vivo study. Therefore, ACP has been incorporated within biodegradable polymers (e.g. PLLA, PLGA) for manufacturing porous scaffolds for bone and cartilage tissue engineering. It was also found that ACP particles within the polymer composites transferred after a short period of immersion in PBS into bone-like apatite which would potentially facilitate the formation of new bone in vivo and clinical trials.^157^ Recently, CaP powder was manufactured into three-dimensional (3D) porous scaffolds with the aid of additive manufacturing techniques (robocasting; see Figure 7).

### Bioactive coatings

Metallic implants are still commonly used for load-bearing applications such as hip-joint replacement, bone fixation devices (i.e. nails, plates and screws) and tooth sockets.^158^ The lack of the bioactivity and poor bonding with the host bone of the metals had been a challenge for their clinical use. Therefore, coating with bioactive materials such as ACP, HA or other CaPs have been conducted to overcome the bioactivity and biocompatibility complications. HA coating of metallic implants was found to enhance their rate of clinical success to be more than 98%.^163^ Numerous coating methods have been utilised, for example, thermal spray, plasma spraying, electrophoretic and biomimetic deposition.^164^ The presence of ACP was reported in plasma-sprayed HA coating; however, the quantity could not be well controlled.^158^

Since magnesium alloys are biodegradable metals, non-toxic and have similar mechanical properties of the cortical bone, they have been considered as attractive candidates for load-bearing biomedical applications. The main complication associated with the use of magnesium implant is the fast degradation in physiological environment. Thus, CaP coatings have been applied to magnesium alloys to enhance their bioactivity, biocompatibility and control their degradation rates. A significant reduction in degradation rates of magnesium alloys was obtained after surface coating with CaP materials.^165^

### Drug and gene delivery

**Figure 8.**
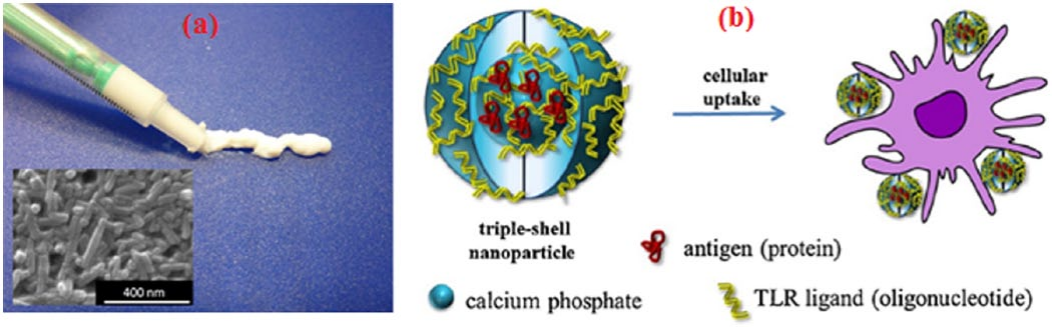
CaP nanoparticles for drug and gene delivery applications; (a) CaP nanorods paste containing DNA encoding fro BMP-7 and VEGF-A for repairing bone defect, Adapted with permission from Chernousova et al.^166^ (b) multi-shell design of CaP nanoparticles loaded with antigen and TLR ligand. Adapted with permission from Sokolova et al.^167^

CaP-based nanoparticles have been explored for targeted drug and gene delivery due to their unique characteristics; similarity to inorganic component of bone, excellent adsorption capability to many biomolecules and proteins and biodegradability in moderate acidic medium (similar to the pH inside lysosome). CaP nanoparticles, with size less than 200 nm, can enter into the cells via endocytosis mechanism and may end up in lysosomes. Consequently, CaP can break down at acidic conditions into phosphate and calcium ions. Phosphate ions are not harmful and amount of calcium ions can be tolerated using moderate quantities of the nanoparticles. Dissolution of CaP nanoparticles after cellular uptake made them a promising competitor to conventional nanoparticles (silica, gold and polymers). CaP nanoparticles loaded with vascular endothelial growth factor (VEGF) and bone morphogenetic proteins (BMPs) were produced in a water-based paste for bone repair purposes through direct injection into the defect (see Figure 8(a)).^161^ CaP nanoparticles can also be used to deliver drugs and biomolecules by producing them with multi-shells architecture (see Figure 8(b)).

### Soft tissue engineering

Due to their ease of preparation into fibres, CaP glass fibres (in particular, phosphate based) were studied for their potential use in muscle and nerve regeneration. A 3D fibrous construct, having the composition of (P_2_O_5_)_62.9_(Al_2_O_3_)_21.9_ZnO_15.2_, supported the proliferation and differentiation of human masseter muscle–derived cell cultures.^9^ CaP glass fibres, based on (P_2_O_5_)_50_(CaO)_30_(Na_2_O)_20-x_(Fe_2_O_3_)_x_ composition where x = 1–5, supported high level of attachment of immortal muscle precursor cell line.^36^

Phosphate glass fibres containing 5–22.5 wt% Fe_2_O_3_ have been used as reinforcing agents in the development of bioabsorbable composites designed for orthopaedic applications. A cortical plug method was used to test the biocompatibility of these glasses; the results showed that no inflammation was observed over periods of up to 5 weeks.^168^ Due to the intriguing ability of phosphate glass fibres to form capillary-like channels during their degradation,^47^ they were used for in situ formation of continuous aligned channels of 30–40 μm in diameter within 3D dense collagen scaffolds to allow for proper diffusion of nutrients and waste products through the constructs.^51^ These constructs therefore maintained an excellent viability of human oral fibroblasts that formed a 3D network.^51^

## Summary

This article aimed to review the various types of biomaterials (i.e. silicate-, borate- and phosphate-based glasses; glass-ceramics; bioceramics; and metals) investigated for in vitro bioactivity evaluation. CaP formation route on the surface of these biomaterials are dependent on the surface activity of the materials (in test medium) and test environment (i.e. ionic concentrations of medium and pH). Materials geometry (i.e. powder, pellets, discs, cubes, blocks), material to medium volume ratio and experimental condition (static or dynamic) are also crucial factors that require consideration during bioactivity test. For example, ISO 23317:2014(E) standard suggests the sample surface area (solid disc/block) to SBF volume ratio (V_SBF_ = 100 × S_a_) to be used at static condition, whereas recently a unified method (modified form of ISO) has been suggested to use of fixed mass (45–90 μm particles) per medium volume ratio (75 mg in 50 mL) with agitation during the bioactivity testing. Apart from these various test parameters, adequate physico-chemical characterisation is necessary to draw conclusive understanding of the nature of deposited CaP phase. Although plenty of ACPs and bone-like apatite materials have already shown their potential in biomedical applications (dental, bone repair, gene and drug delivery) and also some of them are already available in the market, some factors such as possible experimental mistakes including evolution of the substrate in medium, contamination within medium by microorganisms and residual presence of precursor phases are responsible for experimental failure of apatite formation when investigating a new bioactive material, which still warrant further research and validation.

## References

[1] Zhu B Lu Q Yin J Alignment of osteoblast-like cells and cell-produced collagen matrix induced by nanogrooves. Tissue Eng 2005; 11: 825–834. 15998222 10.1089/ten.2005.11.825

[2] Riboldi SA Sadr N Pigini L Skeletal myogenesis on highly orientated microfibrous polyesterurethane scaffolds. J Biomed Mater Res A 2008; 84: 1094–1101. 17685407 10.1002/jbm.a.31534

[3] Johnson WE Wootton A El Haj A Topographical guidance of intervertebral disc cell growth in vitro: towards the development of tissue repair strategies for the anulus fibrosus. Eur Spine J 2006; 15: 389–396. 10.1007/s00586-006-0125-9 PMC2335384 16688474

[4] Her GJ Wu H-C Chen M-H Control of three-dimensional substrate stiffness to manipulate mesenchymal stem cell fate toward neuronal or glial lineages. Acta Biomater 2013; 9: 5170–5180. 23079022 10.1016/j.actbio.2012.10.012

[5] Bryant SJ Nicodemus GD Villanueva I. Designing 3D photopolymer hydrogels to regulate biomechanical cues and tissue growth for cartilage tissue engineering. Pharm Res 2008; 25: 2379–2386. 18509600 10.1007/s11095-008-9619-y

[6] Kokubo T Takadama H. How useful is SBF in predicting in vivo bone bioactivity? Biomaterials 2006; 27: 2907–2915. 16448693 10.1016/j.biomaterials.2006.01.017

[7] Fu Q Rahaman MN Bal BS In vivo evaluation of 13-93 bioactive glass scaffolds with trabecular and oriented microstructures in a subcutaneous rat implantation model. J Biomed Mater Res A 2010; 95: 235–244. 20574983 10.1002/jbm.a.32827

[8] Rahaman MN Day DE Sonny Bal B Bioactive glass in tissue engineering. Acta Biomater 2011; 7: 2355–2373. 21421084 10.1016/j.actbio.2011.03.016 PMC3085647

[9] Shah R Sinanan ACM Knowles JC Craniofacial muscle engineering using a 3-dimensional phosphate glass fibre construct. Biomaterials 2005; 26: 1497–1505. 15522751 10.1016/j.biomaterials.2004.04.049

[10] Moioli EK Clark PA Xin X Matrices and scaffolds for drug delivery in dental, oral and craniofacial tissue engineering. Adv Drug Deliv Rev 2007; 59: 308–324. 17499385 10.1016/j.addr.2007.03.019 PMC4035021

[11] Sepulveda P Jones JR Hench LL. In vitro dissolution of melt-derived 45S5 and sol-gel derived 58S bioactive glasses. J Biomed Mater Res 2002; 61: 301–311. 12007211 10.1002/jbm.10207

[12] Braun S Rappoport S Zusman R Biochemically active sol-gel glasses: the trapping of enzymes. Mater Lett 2007; 61: 2843–2846.

[13] Jacques L Thibaud C Cécile R. Encapsulation of biomolecules in silica gels. J Phys Condens Matter 2001; 13: R673.

[14] Blaker JJ Nazhat SN Boccaccini AR. Development and characterisation of silver-doped bioactive glass-coated sutures for tissue engineering and wound healing applications. Biomaterials 2004; 25: 1319–1329. 14643606 10.1016/j.biomaterials.2003.08.007

[15] Neel EA Ahmed I Pratten J Characterisation of antibacterial copper releasing degradable phosphate glass fibres. Biomaterials 2005; 26: 2247–2254. 15585226 10.1016/j.biomaterials.2004.07.024

[16] Gorustovich AA Roether JA Boccaccini AR. Effect of bioactive glasses on angiogenesis: a review of in vitro and in vivo evidences. Tissue Eng Part B Rev 2010; 16: 199–207. 19831556 10.1089/ten.TEB.2009.0416

[17] Day RM. Bioactive glass stimulates the secretion of angiogenic growth factors and angiogenesis in vitro. Tissue Eng 2005; 11: 768–777. 15998217 10.1089/ten.2005.11.768

[18] Fu Q Saiz E Tomsia AP. Bioinspired strong and highly porous glass scaffolds. Adv Funct Mater 2011; 21: 1058–1063. 21544222 10.1002/adfm.201002030 PMC3085453

[19] Yan X Yu C Zhou X Highly ordered mesoporous bioactive glasses with superior in vitro bone-forming bioactivities. Angew Chem Int Ed Engl 2004; 43: 5980–5984. 15547911 10.1002/anie.200460598

[20] Ghosh SK Nandi SK Kundu B In vivo response of porous hydroxyapatite and β-tricalcium phosphate prepared by aqueous solution combustion method and comparison with bioglass scaffolds. J Biomed Mater Res B Appl Biomater 2008; 86: 217–227. 18161811 10.1002/jbm.b.31009

[21] Zhang X Zeng D Li N Functionalized mesoporous bioactive glass scaffolds for enhanced bone tissue regeneration. Sci Rep 2016; 6: 19361. 26763311 10.1038/srep19361 PMC4725866

[22] López-Noriega A Arcos D Vallet-Regí M. Functionalizing mesoporous bioglasses for long-term anti-osteoporotic drug delivery. Chemistry 2010; 16: 10879–10886. 20661959 10.1002/chem.201000137

[23] Nunzi Conti G Chiasera A Ghisa L Spectroscopic and lasing properties of Er^3+^-doped glass microspheres. J Non Cryst Solids 2006; 352: 2360–2363.

[24] Lakhkar NJ Park J-H Mordan NJ Titanium phosphate glass microspheres for bone tissue engineering. Acta Biomater 2012; 8: 4181–4190. 22835676 10.1016/j.actbio.2012.07.023

[25] Dong CH Xiao YF Han ZF Low-threshold microlaser in Er:Yb phosphate glass coated microsphere. IEEE Photonics Technol Lett 2008; 20: 342–344.

[26] Blaker JJ Knowles JC Day RM. Novel fabrication techniques to produce microspheres by thermally induced phase separation for tissue engineering and drug delivery. Acta Biomater 2008; 4: 264–272. 18032120 10.1016/j.actbio.2007.09.011

[27] Sene FF Martinelli JR Okuno E. Synthesis and characterization of phosphate glass microspheres for radiotherapy applications. J Non Cryst Solids 2008; 354: 4887–4893.

[28] Peitl Filho O La Torre GP Hench LL Effect of crystallization on apatite-layer formation of bioactive glass 45S5. J Biomed Mater Res 1996; 30: 509–514. 8847359 10.1002/(SICI)1097-4636(199604)30:4<509::AID-JBM9>3.0.CO;2-T

[29] Hench LL Andersson Ö. Bioactive glasses. In: Hench LL Wilson J (eds) An introduction to bioceramics. Singapore: World Scientific Publishing, 1993, pp. 41–62.

[30] Li R Clark AE Hench LL Effects of structure and surface area on bioactive powders by sol-gel process. In: Hench LL West JK (eds) Chemical processing of advanced materials, by, J. Wiley and Sons, Inc New York, 1992, pp. 627–633.

[31] Haro Durand LA Vargas GE Romero NM Angiogenic effects of ionic dissolution products released from a boron-doped 45S5 bioactive glass. J Mater Chem B Mater Biol Med 2015; 3: 1142–1148. 10.1039/c4tb01840k 32261993

[32] Munukka E Leppäranta O Korkeamäki M Bactericidal effects of bioactive glasses on clinically important aerobic bacteria. J Mater Sci Mater Med 2008; 19: 27–32. 17569007 10.1007/s10856-007-3143-1

[33] Salih V Franks K James M Development of soluble glasses for biomedical use part II: the biological response of human osteoblast cell lines to phosphate-based soluble glasses. J Mater Sci Mater Med 2000; 11: 615–620. 15348085 10.1023/a:1008901612674

[34] Abou Neel EA O’Dell LA Smith ME Processing, characterisation, and biocompatibility of zinc modified metaphosphate based glasses for biomedical applications. J Mater Sci Mater Med 2008; 19: 1669–1679. 18060479 10.1007/s10856-007-3313-1

[35] Ahmed I Abou Neel EA Valappil SP The structure and properties of silver-doped phosphate-based glasses. J Mater Sci 2007; 42: 9827–9835.

[36] Ahmed I Collins CA Lewis MP Processing, characterisation and biocompatibility of iron-phosphate glass fibres for tissue engineering. Biomaterials 2004; 25: 3223–3232. 14980417 10.1016/j.biomaterials.2003.10.013

[37] Abou Neel EA Chrzanowski W Knowles JC. Effect of increasing titanium dioxide content on bulk and surface properties of phosphate-based glasses. Acta Biomater 2008; 4: 523–534. 18249043 10.1016/j.actbio.2007.11.007

[38] Lakhkar NJ Abou Neel EA Salih V Strontium oxide doped quaternary glasses: effect on structure, degradation and cytocompatibility. J Mater Sci Mater Med 2009; 20: 1339. 19132501 10.1007/s10856-008-3688-7

[39] Valappil SP Pickup DM Carroll DL Effect of silver content on the structure and antibacterial activity of silver-doped phosphate-based glasses. Antimicrob Agents Chemother 2007; 51: 4453–4461. 17908949 10.1128/AAC.00605-07 PMC2168012

[40] Abou Neel EA O’Dell LA Chrzanowski W Control of surface free energy in titanium doped phosphate based glasses by co-doping with zinc. J Biomed Mater Res B Appl Biomater 2009; 89: 392–407. 18837445 10.1002/jbm.b.31227

[41] Bitar M Salih V Knowles JC Iron-phosphate glass fiber scaffolds for the hard-soft interface regeneration: the effect of fiber diameter and flow culture condition on cell survival and differentiation. J Biomed Mater Res A 2008; 87: 1017–1026. 18257069 10.1002/jbm.a.31855

[42] Hoppe U. A structural model for phosphate glasses. J Non Cryst Solids 1996; 195: 138–147.

[43] Bae B-S Weinberg MC. Oxidation–reduction equilibrium in copper phosphate glass melted in air. J Am Ceram Soc 1991; 74: 3039–3045.

[44] Abou Neel EA Chrzanowski W Valappil SP Doping of a high calcium oxide metaphosphate glass with titanium dioxide. J Non Cryst Solids 2009; 355: 991–1000.

[45] Abou Neel EA Knowles JC Physical and biocompatibility studies of novel titanium dioxide doped phosphate-based glasses for bone tissue engineering applications. J Mater Sci Mater Med 2008; 19: 377–386. 17607512 10.1007/s10856-007-3079-5

[46] Abou Neel EA Mizoguchi T Ito M In vitro bioactivity and gene expression by cells cultured on titanium dioxide doped phosphate-based glasses. Biomaterials 2007; 28: 2967–2977. 17412416 10.1016/j.biomaterials.2007.03.018

[47] Abou Neel EA Young AM Nazhat SN A facile synthesis route to prepare microtubes from phosphate glass fibres. Adv Mater 2007; 19: 2856–2862.

[48] Guedes JC Park J-H Lakhkar NJ TiO_2_-doped phosphate glass microcarriers: a stable bioactive substrate for expansion of adherent mammalian cells. J Biomater Appl 2013; 28: 3–11. 22935537 10.1177/0885328212459093 PMC4107757

[49] Vitale-Brovarone C Novajra G Lousteau J Phosphate glass fibres and their role in neuronal polarization and axonal growth direction. Acta Biomater 2012; 8: 1125–1136. 22134161 10.1016/j.actbio.2011.11.018

[50] Abou Neel EA Pickup DM Valappil SP Bioactive functional materials: a perspective on phosphate-based glasses. J Mater Chem B Mater Biol Med 2009; 19: 690–701.

[51] Nazhat SN Abou Neel EA Kidane A Controlled microchannelling in dense collagen scaffolds by soluble phosphate glass fibers. Biomacromolecules 2006; 8: 543–551. 10.1021/bm060715f 17291078

[52] Yao A Wang D Huang W In vitro bioactive characteristics of borate-based glasses with controllable degradation behavior. J Am Ceram Soc 2007; 90: 303–306.

[53] Toker H Ozdemir H Balci Yuce H The effect of boron on alveolar bone loss in osteoporotic rats. J Dent Sci 2016; 11: 331–337. 10.1016/j.jds.2016.03.011 PMC6395371 30894992

[54] Liu X Huang W Fu H Bioactive borosilicate glass scaffolds: improvement on the strength of glass-based scaffolds for tissue engineering. J Mater Sci Mater Med 2008; 20: 365–372. 18807266 10.1007/s10856-008-3582-3

[55] Marion NW Liang W Liang W Borate glass supports the in vitro osteogenic differentiation of human mesenchymal stem cells. Mech Adv Mater Struct 2005; 12: 239–246.

[56] Fu Q Rahaman MN Bal BS Silicate, borosilicate, and borate bioactive glass scaffolds with controllable degradation rate for bone tissue engineering applications. II.In vitro and in vivo biological evaluation. J Biomed Mater Res A 2010; 95: 172–179. 20540099 10.1002/jbm.a.32823

[57] Liu X Xie Z Zhang C Bioactive borate glass scaffolds: in vitro and in vivo evaluation for use as a drug delivery system in the treatment of bone infection. J Mater Sci Mater Med 2010; 21: 575–582. 19830527 10.1007/s10856-009-3897-8

[58] Brown RF Rahaman MN Dwilewicz AB Effect of borate glass composition on its conversion to hydroxyapatite and on the proliferation of MC3T3-E1 cells. J Biomed Mater Res A 2009; 88: 392–400. 18306284 10.1002/jbm.a.31679

[59] Kretlow JD Young S Klouda L Injectable biomaterials for regenerating complex craniofacial tissues. Adv Mater 2009; 21: 3368–3393. 19750143 10.1002/adma.200802009 PMC2742469

[60] Burguera EF Xu HHK Sun L. Injectable calcium phosphate cement: effects of powder-to-liquid ratio and needle size. J Biomed Mater Res B Appl Biomater 2008; 84: 493–502. 17635038 10.1002/jbm.b.30896 PMC2652762

[61] Vorndran E Geffers M Ewald A Ready-to-use injectable calcium phosphate bone cement paste as drug carrier. Acta Biomater 2013; 9: 9558–9567. 23954526 10.1016/j.actbio.2013.08.009

[62] Turner TM Urban RM Singh K Vertebroplasty comparing injectable calcium phosphate cement compared with polymethylmethacrylate in a unique canine vertebral body large defect model. Spine J 2008; 8: 482–487. 18455113 10.1016/j.spinee.2006.12.007

[63] Boccaccini AR Chen Q Lefebvre L Sintering, crystallisation and biodegradation behaviour of Bioglass-derived glass-ceramics. Faraday Discuss 2007; 136: 27–44; discussion 107–123. 17955801 10.1039/b616539g

[64] Zanotto ED. A bright future for glass-ceramics. Am Ceram Soc Bull 2010; 89: 19–27.

[65] Park J Ozturk A. Bioactivity of apatite-wollastonite glass-ceramics produced by melting casting. Surf Rev Lett 2013; 20: 1350010.

[66] Thompson ID Hench LL. Mechanical properties of bioactive glasses, glass-ceramics and composites. Proc IMechE, Part H: J Engineering in Medicine 1998; 212: 127–136. 10.1243/0954411981533908 9612004

[67] Magallanes-Perdomo M Luklinska ZB De Aza AH Bone-like forming ability of apatite–wollastonite glass ceramic. J Eur Ceram Soc 2011; 31: 1549–1561.

[68] Vitale-Brovarone C Verné E Robiglio L Development of glass–ceramic scaffolds for bone tissue engineering: characterisation, proliferation of human osteoblasts and nodule formation. Acta Biomater 2007; 3: 199–208. 17085090 10.1016/j.actbio.2006.07.012

[69] Fujita H Iida H Ido K Porous apatite-wollastonite glass-ceramic as an intramedullary plug. J Bone Joint Surg Br 2000; 82: 614–618. 10855893 10.1302/0301-620x.82b4.9739

[70] Long Q Zhou D-L Zhang X Surface modification of apatite-wollastonite glass ceramic by synthetic coupling agent. Front Mater Sci 2014; 8: 157–164.

[71] Zhu P Masuda Y Koumoto K. The effect of surface charge on hydroxyapatite nucleation. Biomaterials 2004; 25: 3915–3921. 15020168 10.1016/j.biomaterials.2003.10.022

[72] Kokubo T Pattanayak DK Yamaguchi S Positively charged bioactive Ti metal prepared by simple chemical and heat treatments. J R Soc Interface 2010; 7: S503–S513. 20444711 10.1098/rsif.2010.0129.focus PMC3024574

[73] Huang W Rahaman MN Day DE Mechanisms for converting bioactive silicate, borate, and borosilicate glasses to hydroxyapatite in dilute phosphate solution. Phys Chem Glasses 2006; 47: 647–658. 10.1007/s10856-006-9220-z 16770542

[74] Huang W Day DE Kittiratanapiboon K Kinetics and mechanisms of the conversion of silicate (45S5), borate, and borosilicate glasses to hydroxyapatite in dilute phosphate solutions. J Mater Sci Mater Med 2006; 17: 583–596. 16770542 10.1007/s10856-006-9220-z

[75] Geng Z Cui Z Li Z Synthesis, characterization and the formation mechanism of magnesium- and strontium-substituted hydroxyapatite. J Mater Chem B Mater Biol Med 2015; 3: 3738–3746. 10.1039/c4tb02148g 32262848

[76] Hench LL. The story of Bioglass^®^. J Mater Sci Mater Med 2006; 17: 967–978. 17122907 10.1007/s10856-006-0432-z

[77] Hench LL Wilson J. Surface-active biomaterials. Science 1984; 226: 630–636. 6093253 10.1126/science.6093253

[78] Fujibayashi S Neo M Kim H-M A comparative study between in vivo bone ingrowth and in vitro apatite formation on Na_2_O-CaO-SiO_2_ glasses. Biomaterials 2003; 24: 1349–1356. 12527276 10.1016/s0142-9612(02)00511-2

[79] Hench LL. Bioceramics: from concept to clinic. J Am Ceram Soc 1991; 74: 1487–1510.

[80] Clark AE Pantano CG Hench LL. Auger spectroscopic analysis of bioglass corrosion films. J Am Ceram Soc 1976; 59: 37–39.

[81] Gunawidjaja PN Mathew R Lo AYH Local structures of mesoporous bioactive glasses and their surface alterations in vitro: inferences from solid-state nuclear magnetic resonance. Philos Trans R Soc A Math Phys Eng Sci 2012; 370: 1376–1399. 10.1098/rsta.2011.0257 PMC3270387 22349247

[82] Lankford K Letourneau P. Evidence that calcium may control neurite outgrowth by regulating the stability of actin filaments. J Cell Biol 1989; 109: 1229–1243. 2504729 10.1083/jcb.109.3.1229 PMC2115760

[83] Ostomel TA Shi Q Tsung CK Spherical bioactive glass with enhanced rates of hydroxyapatite deposition and hemostatic activity. Small 2006; 2: 1261–1265. 17192971 10.1002/smll.200600177

[84] Chen Q Miyaji F Kokubo T Apatite formation on PDMS-modified CaO-SiO_2_-TiO_2_ hybrids prepared by sol-gel process. Biomaterials 1999; 20: 1127–1132. 10382828 10.1016/s0142-9612(99)00015-0

[85] Rude RK Gruber HE Norton HJ Bone loss induced by dietary magnesium reduction to 10% of the nutrient requirement in rats is associated with increased release of substance P and tumor necrosis factor-α. J Nutr 2004; 134: 79–85. 14704297 10.1093/jn/134.1.79

[86] Rude R Gruber H Wei L Magnesium deficiency: effect on bone and mineral metabolism in the mouse. Calcif Tissue Int 2003; 72: 32–41. 12370796 10.1007/s00223-001-1091-1

[87] Yamasaki Y Yoshida Y Okazaki M Synthesis of functionally graded MgCO_3_ apatite accelerating osteoblast adhesion. J Biomed Mater Res 2002; 62: 99–105. 12124791 10.1002/jbm.10220

[88] Yamasaki Y Yoshida Y Okazaki M Action of FGMgCO_3_Ap-collagen composite in promoting bone formation. Biomaterials 2003; 24: 4913–4920. 14559004 10.1016/s0142-9612(03)00414-9

[89] Zreiqat H Howlett C Zannettino A Mechanisms of magnesium-stimulated adhesion of osteoblastic cells to commonly used orthopaedic implants. J Biomed Mater Res 2002; 62: 175–184. 12209937 10.1002/jbm.10270

[90] Okuma T Magnesium and bone strength. Nutrition 2001; 17: 679–680. 11448600 10.1016/s0899-9007(01)00551-2

[91] Oliveira J Correia R Fernandes M. Surface modifications of a glass and a glass-ceramic of the MgO-3CaO·P_2_O_5_-SiO_2_ system in a simulated body fluid. Biomaterials 1995; 16: 849–854. 8527600 10.1016/0142-9612(95)94146-c

[92] Liang W Rüssel C Day DE Bioactive comparison of a borate, phosphate and silicate glass. J Mater Res 2006; 21: 125–131.

[93] De Aza PN Guitian F De Aza S. Bioactivity of wollastonite ceramics: in vitro evaluation. Scripta Metall Mater 1994; 31: 1001–1005.

[94] De Aza PN Luklinska Z Anseau M Morphological studies of pseudowollastonite for biomedical application. J Microsc 1996; 182: 24–31. 8632444 10.1111/j.1365-2818.1996.tb04794.x

[95] De Aza PN Guitian F De Aza S. Bioeutectic: a new ceramic material for human bone replacement. Biomaterials 1997; 18: 1285–1291. 9307217 10.1016/s0142-9612(97)00063-x

[96] De Aza PN Luklinska Z Anseau M Reactivity of a wollastonite-tricalcium phosphate Bioeutectic^®^ ceramic in human parotid saliva. Biomaterials 2000; 21: 1735–1741. 10905455 10.1016/s0142-9612(00)00058-2

[97] Liu X Ding C Chu PK. Mechanism of apatite formation on wollastonite coatings in simulated body fluids. Biomaterials 2004; 25: 1755–1761. 14738838 10.1016/j.biomaterials.2003.08.024

[98] Xin R Leng Y Chen J A comparative study of calcium phosphate formation on bioceramics in vitro and in vivo. Biomaterials 2005; 26: 6477–6486. 15992923 10.1016/j.biomaterials.2005.04.028

[99] Dorozhkina EI Dorozhkin SV. Surface mineralisation of hydroxyapatite in modified simulated body fluid (mSBF) with higher amounts of hydrogencarbonate ions. Colloids Surf A Physicochem Eng Asp 2002; 210: 41–48.

[100] Pattanayak DK Yamaguchi S Matsushita T Apatite-forming ability of titanium in terms of pH of the exposed solution. J R Soc Interface 2012; 9: 2145–2155. 22417910 10.1098/rsif.2012.0107 PMC3405753

[101] Kokubo T Miyaji F Kim H-M Spontaneous formation of bonelike apatite layer on chemically treated titanium metals. J Am Ceram Soc 1996; 79: 1127–1129.

[102] Kim H-M Miyaji F Kokubo T Preparation of bioactive Ti and its alloys via simple chemical surface treatment. J Biomed Mater Res 1996; 32: 409–417. 8897146 10.1002/(SICI)1097-4636(199611)32:3<409::AID-JBM14>3.0.CO;2-B

[103] Kim HM Miyaji F Kokubo T Graded surface structure of bioactive titanium prepared by chemical treatment. J Biomed Mater Res 1999; 45: 100–107. 10397963 10.1002/(sici)1097-4636(199905)45:2<100::aid-jbm4>3.0.co;2-0

[104] Wei M Kim H-M Kokubo T Optimising the bioactivity of alkaline-treated titanium alloy. Mater Sci Eng C Mater Biol Appl 2002; 20: 125–134.

[105] Jonášová L Müller FA Helebrant A Biomimetic apatite formation on chemically treated titanium. Biomaterials 2004; 25: 1187–1194. 14643592 10.1016/j.biomaterials.2003.08.009

[106] Yousefpour M Afshar A Chen J Bioactive layer formation on alkaline-acid treated titanium in simulated body fluid. Mater Design 2007; 28: 2154–2159.

[107] Kasuga T Kondo H Nogami M. Apatite formation on TiO_2_ in simulated body fluid. J Cryst Growth 2002; 235: 235–240.

[108] Rey C Combes C Drouet C Bone mineral: update on chemical composition and structure. Osteoporos Int 2009; 20: 1013–1021. 19340505 10.1007/s00198-009-0860-y PMC2760485

[109] Kasuga T Hattori T Niinomi M. Phosphate glasses and glass-ceramics for biomedical applications. Phosphorus Res Bull 2012; 26: 8–15.

[110] Rajkumar G Rajendran V Aravindan S. Role of MgO on the HAp forming ability in phosphate based glasses. Ceram Int 2012; 38: 3781–3790.

[111] Kasuga T Hosoi Y Nogami M Apatite formation on calcium phosphate invert glasses in simulated body fluid. J Am Ceram Soc 2001; 84: 450–452.

[112] Rajendran V Rajkumar G Aravindan S Analysis of physical properties and hydroxyapatite precipitation in vitro of TiO_2_-containing phosphate-based glass systems. J Am Ceram Soc 2010; 93: 4053–4060.

[113] Nan Y Lee WE James PF. Crystallization behavior of CaO–P_2_O_5_ glass with TiO_2_, SiO_2_, and Al_2_O_3_ additions. J Am Ceram Soc 1992; 75: 1641–1647.

[114] Kasuga T Hosoi Y Nogami M Biomimetic apatite formation on calcium phosphate invert glasses. Phosphorus Res Bull 2001; 12: 39–44.

[115] Gerhardt L-C Boccaccini AR. Bioactive glass and glass-ceramic scaffolds for bone tissue engineering. Materials 2010; 3: 3867–3910. 10.3390/ma3073867 PMC5445790 28883315

[116] Kokubo T Kim H-M Kawashita M. Novel bioactive materials with different mechanical properties. Biomaterials 2003; 24: 2161–2175. 12699652 10.1016/s0142-9612(03)00044-9

[117] Drouet C. Apatite formation: why it may not work as planned, and how to conclusively identify apatite compounds. Biomed Res Int 2013; 2013: 490946. 23984373 10.1155/2013/490946 PMC3745891

[118] Elliott JC. Structure and chemistry of the apatites and other calcium orthophosphates. Amsterdam: Elsevier, 2013.

[119] Videau J-J Portier J Piriou B. Raman spectroscopic studies of fluorophosphate glasses. J Non Cryst Solids 1982; 48: 385–392.

[120] Helebrant A Jonasova L Sanda L. The influence of simulated body fluid composition on carbonated hydroxyapatite formation. Ceramics 2002; 46: 9–14.

[121] Kokubo T Kushitani H Sakka S Solutions able to reproduce in vivo surface-structure changes in bioactive glass-ceramic A-W. J Biomed Mater Res 1990; 24: 721–734. 2361964 10.1002/jbm.820240607

[122] Varila L Fagerlund S Lehtonen T Surface reactions of bioactive glasses in buffered solutions. J Eur Ceram Soc 2012; 32: 2757–2763.

[123] Abo-Naf SM Khalil E-SM El-Sayed E-SM In vitro bioactivity evaluation, mechanical properties and microstructural characterization of Na_2_O-CaO-B_2_O_3_-P_2_O_5_ glasses. Spectrochim Acta A Mol Biomol Spectrosc 2015; 144: 88–98. 25748986 10.1016/j.saa.2015.02.076

[124] Turdean-Ionescu C Stevensson B Grins J Composition-dependent in vitro apatite formation at mesoporous bioactive glass-surfaces quantified by solid-state NMR and powder XRD. RSC Adv 2015; 5: 86061–86071.

[125] Bui X Nguyen V Le T ‘In vitro’ apatite formation on the surface of bioactive glass. Glass Phys Chem 2013; 39: 64–66.

[126] Lööf J Svahn F Jarmar T A comparative study of the bioactivity of three materials for dental applications. Dent Mater 2008; 24: 653–659. 17727942 10.1016/j.dental.2007.06.028

[127] Al-Noaman A Rawlinson SC Hill RG. The role of MgO on thermal properties, structure and bioactivity of bioactive glass coating for dental implants. J Non Cryst Solids 2012; 358: 3019–3027.

[128] Kim H-M Himeno T Kokubo T Process and kinetics of bonelike apatite formation on sintered hydroxyapatite in a simulated body fluid. Biomaterials 2005; 26: 4366–4373. 15701365 10.1016/j.biomaterials.2004.11.022

[129] Kokubo T Ito S Huang Z Ca, P-rich layer formed on high-strength bioactive glass-ceramic A-W. J Biomed Mater Res 1990; 24: 331–343. 2156869 10.1002/jbm.820240306

[130] Ohtsuki C Kushitani H Kokubo T Apatite formation on the surface of ceravital-type glass-ceramic in the body. J Biomed Mater Res 1991; 25: 1363–1370. 1797808 10.1002/jbm.820251105

[131] Lepry WC Nazhat SN. Highly bioactive sol-gel-derived borate glasses. Chem Mater 2015; 27: 4821–4831.

[132] Liang W Rahaman MN Day DE Bioactive borate glass scaffold for bone tissue engineering. J Non Cryst Solids 2008; 354: 1690–1696.

[133] Theodorou G Goudouri O Kontonasaki E Comparative bioactivity study of 45S5 and 58S bioglasses in organic and inorganic environment. Bioceram Dev Appl 2011; 1: 1–4.

[134] Faure J Balamurugan A Benhayoune H Morphological and chemical characterisation of biomimetic bone like apatite formation on alkali treated Ti6Al4V titanium alloy. Mater Sci Eng C Mater Biol Appl 2009; 29: 1252–1257.

[135] ISO 23317:2014. Implants for surgery: in vitro evaluation for apatite-forming ability of implant materials.

[136] Maçon AL Kim TB Valliant EM A unified in vitro evaluation for apatite-forming ability of bioactive glasses and their variants. J Mater Sci Mater Med 2015; 26: 115. 25665841 10.1007/s10856-015-5403-9

[137] Mei J Shelton R Marquis P. Changes in the elemental composition of Bioglass during its surface development in the presence or absence of proteins. J Mater Sci Mater Med 1995; 6: 703–707.

[138] Radin S Ducheyne P Rothman B The effect of in vitro modeling conditions on the surface reactions of bioactive glass. J Biomed Mater Res 1997; 37: 363–375. 9368141 10.1002/(sici)1097-4636(19971205)37:3<363::aid-jbm7>3.0.co;2-j

[139] Fagerlund S Hupa L Hupa M. Comparison of reactions of bioactive glasses in different aqueous solutions. Ceram Trans 2010; 218: 101.

[140] Rohanová D Boccaccini AR Yunos DM TRIS buffer in simulated body fluid distorts the assessment of glass-ceramic scaffold bioactivity. Acta Biomater 2011; 7: 2623–2630. 21345388 10.1016/j.actbio.2011.02.028

[141] Hlavac J Rohanová D Helebrant A. The effect of tris-buffer on the leaching behaviour of bioactive glass-ceramics. Ceramics 1994; 38: 119–122.

[142] Ogino M Hench LL. Formation of calcium phosphate films on silicate glasses. J Non Cryst Solids 1980; 38: 673–678.

[143] Ogino M Ohuchi F Hench LL. Compositional dependence of the formation of calcium phosphate films on bioglass. J Biomed Mater Res 1980; 14: 55–64. 6244314 10.1002/jbm.820140107

[144] Fujiu T Ogino M. Difference of bond bonding behavior among surface active glasses and sintered apatite. J Biomed Mater Res 1984; 18: 845–859. 6544783 10.1002/jbm.820180714

[145] Pan H Zhao X Darvell BW Apatite-formation ability–predictor of ‘bioactivity’? Acta Biomater 2010; 6: 4181–4188. 20493974 10.1016/j.actbio.2010.05.013

[146] Oyane A Kim H-M Furuya T Preparation and assessment of revised simulated body fluids. J Biomed Mater Res A 2003; 65: 188–195. 12734811 10.1002/jbm.a.10482

[147] Kim HM Miyazaki T Kokubo T Revised simulated body fluid. Key Eng Mat 2001; 192–195: 47–50.

[148] Rohanová D Boccaccini AR Horkavcová D Is non-buffered DMEM solution a suitable medium for in vitro bioactivity tests? J Mater Chem B Mater Biol Med 2014; 2: 5068–5076. 10.1039/c4tb00187g 32261840

[149] Popa A Stan G Husanu M Bioglass implant-coating interactions in synthetic physiological fluids with varying degrees of biomimicry. Int J Nanomedicine 2017; 12: 683–707. 28176941 10.2147/IJN.S123236 PMC5268334

[150] Zhou Z Yi Q Liu X In vitro degradation behaviors of poly-L-lactide/bioactive glass composite materials in phosphate-buffered solution. Polym Bull 2009; 63: 575–586.

[151] Li H Chang J. pH-compensation effect of bioactive inorganic fillers on the degradation of PLGA. Compos Sci Technol 2005; 65: 2226–2232.

[152] Ning J Yao A Wang D Synthesis and in vitro bioactivity of a borate-based bioglass. Mater Lett 2007; 61: 5223–5226.

[153] Rámila A Vallet-Regı M. Static and dynamic in vitro study of a sol-gel glass bioactivity. Biomaterials 2001; 22: 2301–2306. 11456070 10.1016/s0142-9612(00)00419-1

[154] Zhang D Hupa M Aro HT Influence of fluid circulation on in vitro reactivity of bioactive glass particles. Mater Chem Phys 2008; 111: 497–502.

[155] Siriphannon P Kameshima Y Yasumori A Comparative study of the formation of hydroxyapatite in simulated body fluid under static and flowing systems. J Biomed Mater Res 2002; 60: 175–185. 11835173 10.1002/jbm.10056

[156] Strnad J Protivínský J Mazur D Interaction of acid and alkali treated titanium with dynamic simulated body environment. J Therm Anal Calorim 2004; 76: 17–31.

[157] Zhao J Liu Y Sun W-B Amorphous calcium phosphate and its application in dentistry. Chem Cent J 2011; 5: 40. 21740535 10.1186/1752-153X-5-40 PMC3143077

[158] Combes C Rey C. Amorphous calcium phosphates: synthesis, properties and uses in biomaterials. Acta Biomater 2010; 6: 3362–3378. 20167295 10.1016/j.actbio.2010.02.017

[159] Dorozhkin SV Epple M. Biological and medical significance of calcium phosphates. Angew Chem Int Ed Engl 2002; 41: 3130–3146. 12207375 10.1002/1521-3773(20020902)41:17<3130::AID-ANIE3130>3.0.CO;2-1

[160] Dorozhkin SV. Calcium orthophosphates in dentistry. J Mater Sci Mater Med 2013; 24: 1335–1363. 23468163 10.1007/s10856-013-4898-1

[161] Habraken W Habibovic P Epple M Calcium phosphates in biomedical applications: materials for the future? Mater Today 2016; 19: 69–87.

[162] Butscher A Bohner M Doebelin N New depowdering-friendly designs for three-dimensional printing of calcium phosphate bone substitutes. Acta Biomater 2013; 9: 9149–9158. 23891808 10.1016/j.actbio.2013.07.019

[163] Yang Y Kim K-H Ong JL. A review on calcium phosphate coatings produced using a sputtering process – an alternative to plasma spraying. Biomaterials 2005; 26: 327–337. 15262475 10.1016/j.biomaterials.2004.02.029

[164] Gross KA Berndt CC. Biomedical application of apatites. Rev Mineral Geochem 2002; 48: 631–672.

[165] Dorozhkin SV. Calcium orthophosphate coatings on magnesium and its biodegradable alloys. Acta Biomater 2014; 10: 2919–2934. 24607420 10.1016/j.actbio.2014.02.026

[166] Chernousova S Klesing J Soklakova N A genetically active nano-calcium phosphate paste for bone substitution, encoding the formation of BMP-7 and VEGF-A. RSC Adv 2013; 3: 11155–11161.

[167] Sokolova V Knuschke T Kovtun A The use of calcium phosphate nanoparticles encapsulating Toll-like receptor ligands and the antigen hemagglutinin to induce dendritic cell maturation and T cell activation. Biomaterials 2010; 31: 5627–5633. 20417963 10.1016/j.biomaterials.2010.03.067

[168] Lin ST Krebs SL Kadiyala S Development of bioabsorbable glass fibres. Biomaterials 1994; 15: 1057–1061. 7888576

